# Sympathetic Iritis

**Published:** 1887-03

**Authors:** S. Latimer Phillips

**Affiliations:** Savannah, Ga.


### Clinic Reports.

### SYMPATHETIC IRITIS.

BY S. LATIMER PHILLIPS, M. D., SAVANNAH, GA.,

FORMERLY HOUSE SURGEON TO PRESBYTERIAN EYE, EAR AND THROAT CHARITY HOSPITAL, BALTIMORE, MD.

From among my notes upon a large number of cases of sympathetic eye troubles, I have selected the following two, both being of great interest, but from different points of view—the first being peculiarly so, in that the iritis developed some days after the removal of the offending eye to protect its fellow, a rather novel experience with me, and I am inclined to think so with others, as I have seen very few cases reported in my readings. The second case is a good illustration of what danger an eye is exposed to when the other has been lost from a wound through the ciliary region and still retained. The terrible consequences likely to follow point out clearly and plainly to my mind what must or ought to be the plan of treatment pursued for the greatest benefit to your patient and satisfaction to yourself, and in this it ought to be of great interest to us all.

*Case I.*—Mrs. Eliza S., a strong and healthy countrywoman, aged thirty-three years, four years since was struck on the right eye by a rebounding nail. Sight rapidly failed until it was totally gone. From the healing of the wound up to the present time, she has had no pain in the injured eye. Saw patient first on October 11th. Four months previous to this she noticed that the sight in the left eye was failing. She could not see to sew or read comfortably. In reading the letters would run together and the whole page become blurred, causing her to give it up altogether. Even at a distance she could not see objects as readily as before. From the fact that she had no pain in the right eye, even on pressure, which was now an atrophied and shrunken stump, and that with ophthalmoscope could detect no change in fundus of left, a pair of weak convex glasses were ordered for her to correct the hypermetropia, and she was sent home. In a week she came back, finding that in that time there had been no improvement; her sight was getting worse all the time. She was now advised to get rid of this useless right eye ; she consented, and the stump was enucleated. Everything went on well and the day after the operation she returned home. Up to the fourth day after the operation, the remaining eye seemed to be strengthened ; on that day she began to lose sight rapidly. The family physician was sent for, and on the eleventh day blistered her behind the ear. On the eleventh day after the removal of the eye, she began to have pain in the remaining one. Her husband, now becoming alarmed, brought her to town again, when I saw her nineteen days after the enucleation. I found the left eye in the following condition: Much injection of ciliary region; eye painful and neuralgia about brow; iris swollen and discolored, with pupil responding slowly to light; cornea somewhat hazy; media so cloudy that details of fundus could not be made out; vision reduced to counting fingers at one foot. She was immediately put upon hydrarg. bichloride, gr. $\frac{1}{16}$ in solution four times daily. Locally, atrophine sulphate, gr. iv. to aq. ℥i, to be instilled in eye six times per day. On following day pupil fully dilated, and patient in much better condition. She continued to improve under this treatment, and in a few days was sent home free from danger and sight improving each day.

Why this trouble should develop at such a late day I cannot say. Often and again a diseased eye is removed when its fellow is in an irritable state, and often in the earlier stages of inflammation, and the consequence is, as soon as the exciting cause is taken away, the remaining eye begins to improve, but where the disease develops in the good eye a number of days after the removal of the bad one is to me a new phase.

*II.*—Willie L., æt. 7, two months before I saw him, was struck in the left eye, making a wound in ciliary region. The family doctor was called in and treated the case, and for three weeks everything seemed to go on well to the gratification of parents as well as doctor. At the end of this time the eye became very much inflamed and painful and sight rapidly failed. Notwithstanding the mild course of treatment still kept up by the physician in attendance, the eye was now blind. At the beginning of the first week, dating from the time of the injury, the right eye became weak, suffering much from photophobia and lachrymation. The mother, now becoming alarmed, wished to consult a specialist, but the father still clung to the family doctor, and begged the mother to wait a little longer in the hope that in a few days the eye would begin to improve, and it was put off from day to day in this vain hope; still no improvement came; the sight was rapidly diminishing, but the parents hardly noticed this, as the eye was kept bandaged all the time to protect it from the light. The mother now insisted that the child should be brought down to the city to see an "eye doctor." When I saw the little fellow, both eyes were covered by numberless handkerchiefs, as he could not bear a ray of light scarcely. The child was quite brave under all his suffering and did not complain. When I would attempt to separate the lids the tears would gush out in abundance. After a little I got a look in the eyes, and found the left entirely gone, with not even light appreciable, which was all he had in the right. Pupil closed up almost entirely under chloroform; left eye was removed, and strong solution of atrophine used in right in the hope of breaking up, if possible, the adhesion which had taken place between iris and outer lens capsule. The following day the eye felt better, was less watering, but no improvement in sight. The patient now returned home with his almost grief-stricken parents, with advice to continue the atrophine until all inflammation was allayed, but with very little hope for restoration of sight.

I report this case, not that it is rare or unique, but that it is an example of one of the most common eye injuries, and when neglected will often result most unhappily to the patient, changing and marring the whole course of his future life. If this little patient could have been seen in time by one whose familiarity with the nature of these troubles would have entitled him to a correct judgment, the offending and useless eye would have been removed, and in this manner the other protected, thus saving the child *one* of those valuable organs of vision without which we are of little use to the world. As it is, it is most likely he will become a burden upon his family or friends, or else be one more added to the already too large number of blind children in the various blind institutions of the country.

My experience has taught me that an eye, wounded as in the case above, cannot be trifled with, and I have made it a rule to advise persons consulting me for a *painful* and *useless* eye—not living in the immediate reach of a specialist—to have it removed as a protection to the good one. If they then fail to accept my advice, and the eye takes on sympathetic trouble, my conscience feels clear, as I have done my duty. They are carrying around with them, metaphorically speaking, a loaded shell, which is liable to take fire at any moment and result most seriously to them.

---

OPIUM-SMOKING.—In an article in the *Indian Medical Gazette*, Surgeon-General Moore defends the practice of opium-smoking, and maintains that it is not only not harmful when indulged in moderately, but that it is even beneficial. The use of opium, he says, may be continued for years without the necessity of increasing the dose, and a moderate opium-smoker is no more likely to become an "opium fiend" than is the temperate user of alcohol to become a drunkard, and even among the confirmed abusers of the drug there are seen none of the distressing effects of alcoholic intoxication. The writer says that the members of the Anti-Opium Society, in their misplaced zeal, would take away from the poor an inexpensive source of enjoyment, a protection from disease, and an alleviation of their sufferings. But perhaps his strongest argument, and a very characteristic one, is that the Anti-Opium Society, if it succeeded in its objects, would diminish the revenue of India by seven or eight million pounds sterling. —*Medical Record.*

